# Impact of lipid profiles and risk factors on all-cause mortality in males and females: insights from a Turkish cohort study

**DOI:** 10.1515/med-2026-1504

**Published:** 2026-08-06

**Authors:** Ugurcan Sayili, Busra Albayrak, Kevser Sak, Aysem Kaya, Osman Mutluhan Ugurel, Mehmet Umut Capar, Aysen Fenercioglu, Gunay Can

**Affiliations:** Department of Public Health, Cerrahpaşa Faculty of Medicine, Istanbul University-Cerrahpaşa, Istanbul, Türkiye; Dulkadiroğlu District Health Directorate, Republic of Türkiye Ministry of Health, Kahramanmaraş, Türkiye; Department of Biochemistry, Institute of Cardiology, Istanbul University-Cerrahpaşa, Istanbul, Türkiye; Department of Biostatistics and Medical Informatics, Cerrahpaşa Faculty of Medicine, Istanbul University-Cerrahpaşa, Istanbul, Türkiye; Department of Internal Medicine, Division of Endocrinology and Metabolism, Cerrahpaşa Faculty of Medicine, Istanbul University-Cerrahpaşa, Istanbul, Türkiye; Department of Family Medicine, Cerrahpaşa Faculty of Medicine, Istanbul University-Cerrahpaşa, Istanbul, Türkiye

**Keywords:** lipid profiles, all-cause mortality, cardiovascular risk factors, Turkish cohort, TEKHARF study

## Abstract

**Objectives:**

This study examined associations between lipid profiles, anthropometric measures, and all-cause mortality in a Turkish adult cohort, stratified by sex.

**Methods:**

This prospective study analyzed 2,378 participants from the TEKHARF cohort over a median follow-up of 256 months (up to 25 years). All-cause mortality was evaluated using sex-specific Cox proportional hazards regression models and Restricted Cubic Spline analysis to assess non-linear associations with baseline lipid parameters, anthropometric measures, and clinical risk factors.

**Results:**

During follow-up, 811 deaths (34.1 %) occurred, with significantly higher mortality in males vs. females (37.5 vs. 30.6 %, p<0.001). Low income, physical inactivity, hypertension, and diabetes were strongly associated with mortality in both sexes. In multivariate models, age, hypertension, and diabetes emerged as the most robust independent predictors of mortality. Although total cholesterol and LDL-C quartiles showed associations with mortality in unadjusted analyses, these became non-significant after adjustment. In males, HDL-C in the third quartile (Q3) was associated with a lower risk of mortality compared to the lowest quartile (HR: 0.759, p=0.045), whereas the highest triglyceride quartile independently predicted mortality only in males (HR: 1.464, p=0.008).

**Conclusions:**

Traditional cardiometabolic risk factors, particularly age, hypertension, and diabetes, were the dominant mortality determinants. Notably, in males, triglycerides demonstrated a sex-specific independent effect on mortality risk, while intermediate HDL-C levels were associated with reduced mortality, emphasizing the importance of comprehensive risk assessment integrating lipid parameters with established clinical factors.

## Introduction

Cardiovascular diseases are among the primary causes of death globally, and understanding lipid profiles for predicting all-cause mortality is crucial, especially since risk factors include elevated low-density lipoprotein cholesterol, hypertension, diabetes, smoking, and a history of cardiovascular disease [1], 2].

The relationship between lipid profiles and all-cause mortality has been the subject of extensive research in recent years. High total cholesterol and low-density lipoprotein (LDL) cholesterol levels have traditionally been associated with increased cardiovascular risk [3], 4]. Associations of these parameters with all-cause mortality are more nuanced. Large-scale epidemiological studies have shown a U-shaped or J-shaped association between LDL-C and HDL-C levels and all-cause mortality, suggesting that both very low and very high LDL-C levels may increase the risk of death [5], 6]. A large-scale prospective cohort research in Denmark has revealed a U-shaped association between LDL-C levels and all-cause mortality in the general population, indicating that both low and high LDL-C concentrations are linked to increased mortality risk [5]. Recent studies have similarly revealed a more complex relationship between HDL-C and mortality outcomes. A meta-analysis involving more than 3.5 million participants showed that the relationship between HDL-C levels and all-cause mortality followed a J-shaped dose-response model rather than a simple inverse relationship [7]. This paradigm shift suggests that both extremely low and extremely high HDL-C concentrations are associated with an increased risk of mortality. These findings fundamentally challenge the notion that “lower LDL and higher HDL-C are always better” and emphasize the importance of maintaining HDL-C levels within an optimal range rather than pursuing to maximize HDL-C levels and minimize LDL-C levels [7].

Similarly, the relationship between triglyceride levels and all-cause mortality has been clarified by recent studies. Huang et al. reported that higher triglyceride levels are associated with an increased risk of all-cause mortality and identified an optimal triglyceride level around 135 mg/dL [8]. In addition, a meta-analysis demonstrated that elevated triglyceride levels are dose-dependently associated with a higher risk of mortality. These findings highlight the importance of maintaining triglyceride concentrations within an optimal range, rather than simply aiming for the lowest possible values [9].

Some studies suggest that the relationship between anthropometric measurements (waist and hip circumference, BMI) and all-cause mortality is opposite to what is expected, which we will examine as risk factors in our study. A meta-analysis in Iran revealed a J-shaped association between waist circumference and all-cause mortality [10]. In a Taiwanese study of 627 elderly participants, the relationship between waist circumference and all-cause mortality was found to be U shaped [11].

Evidence from prospective cohort studies suggests that the relationship between lipid parameters and mortality may differ substantially by sex. Janghorbani et al. demonstrated in a large population-based cohort that serum cholesterol was a statistically significant predictor of cardiovascular disease mortality only in males, while its predictive value was not significant in females after multivariate adjustment – highlighting that cardiovascular risk factors may operate differently across sexes [12]. Similarly, Huang et al. reported that elevated triglyceride levels were independently associated with all-cause mortality, with subgroup analyses revealing that this association was significant in males but not in females [8]. Despite these observations, sex-stratified analyses of lipid-mortality associations remain scarce in non-Western populations, highlighting the need for such investigations in understudied cohorts such as the Turkish adult population.

The Turkish Adult Cardiovascular Disease and Risk Factors Survey (TEKHARF) is a prospective cohort study launched in 1990 to assess cardiometabolic risks in adults and their associations with morbidity and mortality [13]. Prior TEKHARF publications have examined lipid profiles and anthropometric measures primarily in relation to cardiovascular disease outcomes using quartile-based categorical analyses, with sex-stratified approaches revealing different risk thresholds and significant predictors between men and women. However, the association between lipid parameters and all-cause mortality has not been previously evaluated in the TEKHARF cohort. Furthermore, restricted cubic spline (RCS) regression, which allows for the modeling of continuous, non-linear relationships and the identification of specific inflection points, has not been applied in prior TEKHARF analyses. The present study expands upon previous TEKHARF research by systematically evaluating lipid profiles and anthropometric measurements in relation to all-cause mortality, using sex-specific RCS analyses. This provides a more thorough evaluation of risk relationships in this Turkish population.

This study aimed to examine the relationship between lipid profiles (including total cholesterol, LDL-C, HDL-C and triglyceride levels) and anthropometric measures, as well as their respective effects on all-cause mortality rates, in males and females in the Türkiye cohort database.

## Methods

### Design

The Turkish Adult Screening for Heart Disease and Risk Factors (TEKHARF) is a prospective cohort study established to evaluate the cardiometabolic risks of adults and their associations with morbidity and mortality, which started in 1990 [13] Since comprehensive lipid panel measurements were first conducted during the 1998 survey, this period was selected as the baseline for the present analysis. All-cause mortality was evaluated over a follow-up period of up to 25 years (median 256 months).

### Ethical statement

The study was performed in accordance with the ethical principles of the Declaration of Helsinki and was approved by the Ethics Committee of Istanbul University-Cerrahpasa Medical Faculty (Approval Number: 20223/05; Date: 01.04.2023).

### Informed consent

Written informed consent was obtained from all individuals included in this study.

### Data collection and procedures

Data was collected through blood sampling and anthropometric measurements. On the basis of patient self-reports, data on patients’ habits were obtained.

**Figure 1:**
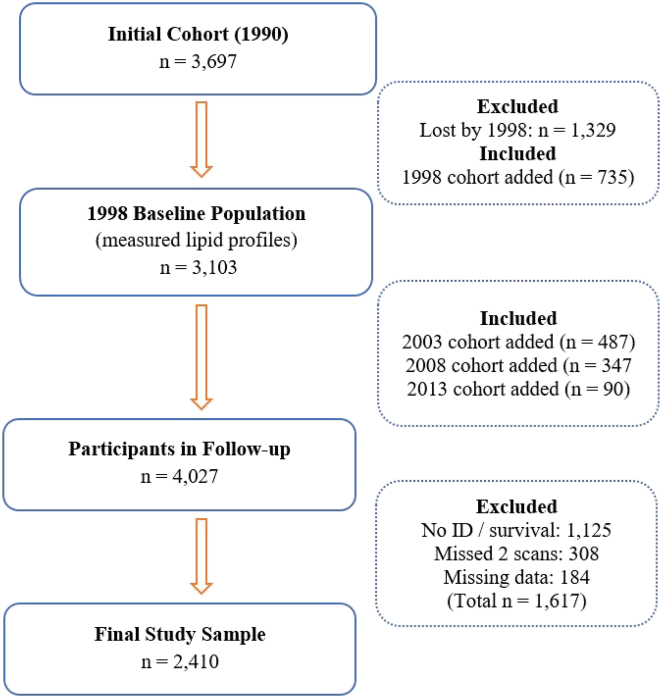
Flow diagram of the study population recruitment and selection process. TEKHARF, Turkish adult risk factor study.

The recruitment and exclusion process of the study participants, starting from the initial 1990 cohort and incorporating subsequent cohorts to reach the final analysis sample of 2,410 individuals, is detailed in the flow diagram (Figure 1).

Between 1998 and 2016, a total of 4,027 participants, with a median age of 52 years, were followed in the TEKHARF cohort, which was established through a sampling method to represent the adult population in Türkiye. Additionally, participants with known malignancy or documented limited life expectancy (less than 6 months) were not included in the original TEKHARF cohort, since the primary objective of the cohort was to investigate long-term cardiovascular risk factors in a generally healthy adult population.

Among these participants, those who did not have a valid Turkish ID number or whose survival status could not be obtained (1,125), who missed 2 consecutive cohort scans (308) and who had substantial missing biochemical and anthropometric measurement data (184) were excluded from our study. However, those with only a few missing values for certain parameters were included to preserve sample size and statistical power. In this regard, 68 participants (32 males, 36 females) with missing BMI data and 2 participants (1 male, 1 female) with missing age data were included in the analysis.

The questionnaires completed by the patients included the patients’ name and surname, TC ID number, year of birth, monthly income level of the family, physical activity levels, and smoking and alcohol habits. According to their answers about smoking, they were classified as never, former or current; according to their alcohol use habits, they were classified as alcohol users or nonusers. The daily physical activity level was assessed with two grades (more active and less active), reflecting the original categorical data collection format of the TEKHARF cohort.

The physical examination included height measurements without shoes and body weight weighing without heavy outer clothing. Body mass index (kg/m^2^) was calculated from the examination findings. Waist circumference was measured with a mesometer while the subject was standing, over the underwear, at the level midway between the lower rib margin and the crista iliaca at the end of mild expiration, and hip circumference was measured at the level of the greater trochanters [13]. Blood pressure was measured with an aneroid sphygmomanometer (Erka, Bad Tölz, Germany) in the sitting position on the right arm, and the mean of two recordings 5 min apart was recorded. Blood samples were collected, spun at 1,000×*g*, shipped to Istanbul and stored at −75 °C until analysis. Serum concentrations of glucose, total cholesterol, fasting triglycerides, low-density lipoprotein (LDL)-cholesterol and high-density lipoprotein (HDL)-cholesterol were determined via a Cobas 500 autoanalyzer (Roche Diagnostic GmbH, Germany).

Blood pressure ≥140/90 mmHg or a history of antihypertensive treatment is considered hypertension. A fasting blood glucose level of 126 mg/dL, a HbA1C of 6.5 %, or the receiving diabetic treatment were considered indicators of diabetes.

### Exposure and outcomes

In our study, the dependent variables were survival status; the independent variables were age, income status, smoking status, physical activity status, chronic disease status (diabetes mellitus, hypertension), anthropometric measurements (waist circumference, hip circumference, BMI), systolic blood pressure, diastolic blood pressure, and lipid profiles (total cholesterol, HDL-C, LDL-C, triglyceride).

The follow-up duration (in months) was calculated for each participant, and all analyses were performed on the basis of the follow-up duration. The baseline for cohort follow-up was defined as the date of participants’ enrollment in the TEKHARF. The survival status of all participants was reviewed on December 4, 2023. For participants who were alive, the study endpoint was December 4, 2023, and for deceased participants, the study endpoint was the date of death.

### Statistical analysis

Statistical analysis was performed using IBM SPSS Statistics ver. 31 (IBM Corp., Armonk, NY, USA). Continuous variables were assessed for normality using visual inspection of histograms and Q-Q plots. Logarithmic transformation was applied to variables with skewed distributions; diastolic blood pressure, total cholesterol, and HDL-cholesterol achieved normal distribution after transformation. Normally distributed variables are presented as mean ± standard deviation (SD), while non-normally distributed variables are expressed as median (minimum–maximum). Categorical variables are reported as frequencies and percentages.

Restricted Cubic Spline (RCS) analyses were conducted in Python (version 3.12) using following libraries; lifelines for survival modeling (CoxPHFitter), patsy for spline basis construction, and SciPy, NumPy, Pandas, and Matplotlib for numerical/statistical computation, data handling, preprocessing, curation, and visualization.

Comparisons of continuous variables between groups were performed using the Independent Samples t-test for normally distributed data and the Mann-Whitney *U* test for non-normally distributed data. Categorical variables were compared using Pearson’s Chi-Square test or Fisher’s Exact test, as appropriate; groups denoted with different superscript letters were significantly different from each other. Lipid parameters were categorized into sex-specific quartiles (Q1–Q4) to evaluate dose-response relationships with all-cause mortality, and differences across quartile groups were assessed using Pearson’s Chi-Square test.

The associations between baseline variables and all-cause mortality were evaluated using Cox proportional hazards regression models, stratified by sex. In the unadjusted model (Model 1), each variable was entered individually. Variables retained in the multivariable Model 2 included age, smoking status, income level, physical activity level, waist circumference, hypertension, and diabetes – all of which are recognized as independent predictors of all-cause mortality and potential confounders in the relationship between lipid profiles and mortality. The independent contribution of each lipid parameter (total cholesterol, HDL-C, LDL-C, and triglycerides) to mortality risk was assessed in separate models (Models 3–6), each incorporating the lipid quartile of interest in addition to all covariates included in Model 2. Results are reported as hazard ratios (HR) with 95 % confidence intervals (CI). Prior to multivariable modeling, we evaluated intercorrelations among anthropometric measures (BMI, waist circumference, and hip circumference). Given the high correlations observed among these variables, we selected waist circumference as the primary anthropometric covariate in adjusted models based on its strongest univariate association with mortality (lowest p-value in preliminary Cox analyses) and its established clinical relevance as a marker of central adiposity and cardiometabolic risk.

To explore potential non-linear relationships between continuous variables and all-cause mortality, RCS analyses were performed using four knots placed at the 5th, 35th, 65th, and 95th percentiles of each variable’s distribution. RCS analyses were conducted in both unadjusted (Model 1) and adjusted (Model 2) frameworks, with the same covariates as described above. The global p-value for the spline term was used to assess the statistical significance of the overall association. A p-value of <0.05 was considered statistically significant.

## Results

### Baseline characteristics

Over a median follow-up of 256 months, we documented 2,410 persons and 821 (34.1 %) cases of all-cause mortality. A comparison of baseline characteristics according to mortality and sex is summarized in Table 1 and Supplementary Table 1. Significant sex-based disparities were observed in lifestyle and clinical profiles: males had a higher prevalence of smoking (48.1 vs. 16.2 %) and alcohol use (18 vs. 0.8 %), while females exhibited higher rates of physical inactivity (74.6 vs. 50.6 %) and higher BMI (p<0.001 for all). During the follow-up period, the all-cause mortality rate was significantly higher in males than in females (37.5 vs. 30.6 %, p<0.001).

**Table 1:** Baseline characteristics of study participants stratified by sex and mortality status.

	Males			Females		
	Alive (n=757)	Death, n=454	p-Value	Alive, n=832	Death, n=367	p-Value
Income, %						

Low	335 (53.5 %)	291 (46.5 %)^a^	**<0.001** ^**d**^	430 (60.6 %)	279 (39.4 %)^a^	**<0.001** ^**d**^
Middle	182 (70.3 %)	77 (29.7 %)^b^		170 (78 %)	48 (22 %)^b^	
High	240 (73.6 %)	86 (26.4 %)^b^		232 (85.3 %)	40 (14.7 %)^b^	

Smoke						

Never	223 (65.2 %)	119 (34.8 %)^a^	**<0.001** ^**d**^	637 (66.6 %)	320 (33.4 %)^a^	**<0.001** ^**d**^
Former	152 (53 %)	135 (47 %)^b^		38 (79.2 %)	10 (20.8 %)^a.b^	
Current	382 (65.6 %)	200 (34.4 %)^a^		157 (80.9 %)	37 (19.1 %)^b^	

Alcohol user						

No	619 (62.3 %)	374 (37.7 %)	0.790^d^	825 (69.4 %)	364 (30.6 %)	1.000^g^
Yes	138 (63.3 %)	80 (36.7 %)		7 (70 %)	3 (30 %)	

Physical activity						

Low	325 (53 %)	288 (47 %)	**<0.001** ^**d**^	579 (64.7 %)	316 (35.3 %)	**<0.001** ^**d**^
More active	432 (72.2 %)	166 (27.8 %)		253 (83.2 %)	51 (16.8 %)	
Waist circumference, cm	93.96 ± 10.53	96.75 ± 10.98	**<0.001** ^**f**^	90.99 ± 12.51	95.32 ± 12.34	**<0.001** ^**f**^
Hip circumference, cm	100.98 ± 7.54	101.6 ± 8.1	0.177^f^	107.42 ± 10.82	108.95 ± 11.74	**0.028** ^**f**^
BMI	26.81 ± 3.9	27.11 ± 4.01	0.212^f^	29.36 ± 5.44	30.08 ± 6.03	0.053^f^
SBP, mmHg	120 (80–207)	135 (85–247)	**<0.001** ^**e**^	125 (80–250)	145 (81–250)	**<0.001** ^**e**^
DBP, mmHg	80 (50–130)	82 (50–147.5)	**<0.001** ^**e**^	80 (48–130)	86 (60–170)	**<0.001** ^**e**^
Total cholesterol, mg/dL^c^	185.6 ± 37.72 181.8 (102.51–343.48)	191.49 ± 40.06 187.93 (95.48–361.58)	**0.014** ^**f**^	191.69 ± 39.37 188.37 (95.95–352)	205.57 ± 40.98 200 (90.45–349.65)	**<0.001** ^**f**^
HDL cholesterol, mg/dL^c^	37.49 ± 11.25 35.32 (13.13–93.55)	38.75 ± 12.46 36.65 (15.12–87.35)	0.113^f^	44.87 ± 12.64; 43 (20–95)	38.75 ± 12.46 36.65 (15.12–87.35)	0.242^f^
LDL cholesterol, mg/dL	114.78 (28.46–226.8)	118.56 (27.79–246.45)	**0.028** ^**e**^	116.99 (23.38–276.42)	125.68 (45–272.9)	**<0.001** ^**e**^
Triglyceride, mg/dL	136.2 (51.59–763)	124.91 (53–637)	0.684^e^	116.64 (52–850)	130.8 (51.59–704.35)	**<0.001** ^**e**^

Hypertension						
Absent	538 (72.9 %)	200 (27.1 %)	**<0.001** ^**d**^	515 (82 %)	113 (18 %)	**<0.001** ^**d**^
Present	219 (46.3 %)	254 (53.7 %)		317 (55.5 %)	254 (44.5 %)	

Diabetes mellitus						

Absent	703 (65.4 %)	372 (34.6 %)	**<0.001** ^**d**^	762 (71.9 %)	298 (28.1 %)	**<0.001** ^**d**^
Present	54 (39.7 %)	82 (60.3 %)		70 (50.4 %)	69 (49.6 %)	

^a,b^Within each sex, categories that do not share a common superscript letter differ significantly from each other. ^c^Logaritmik. ^d^Pearson’s Chi Square test. ^e^Mann Whitney U test. ^f^Independent samples of T-test. ^g^Fisher’s exact test. Bold values indicate statistical significance (p<0.05).

In both sexes, higher mortality rates were associated with low income (46.5 % in males, 39.4 % in females) and physical inactivity (47 % in males, 35.3 % in females) compared to high income (26.4 % in males, 14.7 % in females) and more active groups (27.8 % in males, 16.8 % in females) (p<0.001 for all). The presence of hypertension (53.7 % in males, 44.5 % in females) and diabetes mellitus (60.3 % in males, 49.6 % in females) also showed significant associations with mortality (p<0.001). Deceased participants had significantly higher waist circumference (96.75 ± 10.98 vs. 93.96 ± 10.53 cm in males; 95.32 ± 12.34 vs. 90.99 ± 12.51 cm in females, p<0.001) and higher SBP values (135 vs. 120 mmHg in males; 145 vs. 125 mmHg in females, p<0.001) compared to survivors. Regarding lipid parameters in Table 1, deceased males had significantly higher total cholesterol (191.49 ± 40.06 vs. 185.6 ± 37.72 mg/dL, p=0.014), LDL-cholesterol (118.56 vs. 114.78 mg/dL, p<0.001), and triglyceride levels (136.2 vs. 124.91 mg/dL, p<0.001) than survivors, while HDL-cholesterol levels did not differ significantly (38.75 ± 12.46 vs. 37.49 ± 11.25 mg/dL, p=0.242). In females, deceased participants exhibited higher LDL-cholesterol (125.68 vs. 116.99 mg/dL, p<0.001), triglyceride levels (130.8 vs. 116.64 mg/dL, p<0.001) and total cholesterol (200 vs. 188.37 mg/dL, p<0.001) compared to those who remained alive, whereas HDL-cholesterol (43 vs. 36.65 mg/dL, p=0.242) levels showed no significant difference between the groups. A detailed breakdown of these parameters by age groups (≤52 and >52 years) is provided in Supplementary Table 2.

### Associations between lipid profile quartiles and all-cause mortality

The relationship between lipid quartiles (Q) and mortality (Table 2) revealed that in males, total cholesterol (Q1: 32.6 % vs. Q4: 43.5 %, p=0.043) and LDL-C (Q1: 34.5 % vs. Q4: 45.7 %, p=0.023) quartiles were associated with mortality, while HDL-C (p=0.414) and triglyceride (p=0.283) quartiles showed no significant association. In females, total cholesterol (Q1: 19.7 % vs. Q4: 40.8 %, p<0.001), LDL-C (Q1: 23.3 % vs. Q4: 39.8 %, p<0.001), and triglyceride (Q1: 23.7 % vs. Q4: 38.0 %, p=0.002) quartiles were significantly associated with mortality, while HDL-C quartiles showed no significant association (p=0.057).

**Table 2:** Distribution of all-cause mortality across sex-specific lipid profile quartiles.

Total cholesterol, mg/dL	Males			Total cholesterol, mg/dL	Females		
	Alive	Death	p-Value		Alive	Death	p-Value
	
Q1, <160.8	269 (67.4 %)	130 (32.6 %^)a^	**0.043** ^**d**^	Q1, <168.21	241 (80.3 %)	59 (19.7 %)^a^	**<0.001** ^**d**^
Q2, 160.8–184.9	192 (60.4 %)	126 (39.6 %)^a.b^		Q2, 168.21–192.93	210 (70 %)	90 (30 %)^b^	
Q3, 184.9–210.6	170 (62.7 %)	101 (37.3 %)^a.b^		Q3, 192.93–220.25	204 (68 %)	96 (32 %)^b,c^	
Q4, >210.6	126 (56.5 %)	97 (43.5 %^)b^		Q4, >220.25	177 (59.2 %)	122 (40.8 %)^c^	
HDL cholesterol, mg/dL							
Q1, <29.89	195 (64.4 %)	108 (35.6 %)	0.414^d^	Q1, <36.28	73 (70.9 %)	30 (29.1 %)	0.057^d^
Q2, 29.89–35.64	197 (64.4 %)	109 (35.6 %)		Q2, 36.28–43.68	126 (74.1 %)	44 (25.9 %)	
Q3, 35.64–43.55	188 (62.7 %)	112 (37.3 %)		Q3, 43.68–52.00	236 (73.1 %)	87 (26.9 %)	
Q4, >43.55	177 (58.6 %)	125 (41.4 %)		Q4, >52.00	397 (65.8 %)	206 (34.2 %)	
LDL cholesterol, mg/dL							
Q1, <95.20	226 (65.5 %)	119 (34.5 %)^a^	**0.023** ^**d**^	Q1, <99.10	230 (76.7 %)	70 (23.3 %)^a^	**<0.001** ^**d**^
Q2, 95.20–115.80	200 (63.1 %)	117 (36.9 %)^a,b^		Q2, 99.10–119.58	212 (70.7 %)	88 (29.3 %)^a^	
Q3, 115.80–138.1	197 (65.2 %)	105 (34.8 %)^a,b^		Q3, 119.58–143.81	210 (70 %)	90 (30 %)^a,b^	
Q4, >138.13	134 (54.3 %)	113 (45.7 %)^b^		Q4, >143.81	180 (60.2 %)	119 (39.8 %)^b^	
Triglyceride, mg/dL							
Q1, <92.86	186 (61.4 %)	117 (38.6 %)	0.283^d^	Q1, <85.58	283 (76.3 %)	88 (23.7 %)^a^	**0.002** ^**d**^
Q2, 92.86–134.04	181 (59.7 %)	122 (40.3 %)		Q2, 85.58–120.84	218 (67.5 %)	105 (32.5 %)^a.b^	
Q3, 134.04–196.85	203 (67 %)	100 (33 %)		Q3, 120.84–176.04	189 (68.5 %)	87 (31.5 %)^a,b^	
Q4, >196.85	187 (61.9 %)	115 (38.1 %)		Q4, >176.04	142 (62 %)	87 (38 %)^b^	

^a,b,c^Within each sex and lipid parameter, quartile groups that do not share a common superscript letter differ significantly from each other. ^d^Pearson’s Chi square test. Bold values indicate statistical significance (p<0.05).

### Survival analyses

The independent effects of sociodemographic, clinical, and lipid parameters on all-cause mortality were evaluated using Cox regression models (Table 3). In the unadjusted analysis (Model 1), all primary variables were significantly associated with mortality in both sexes (p<0.001). Age (HR: 1.089 in males, 1.098 in females), low physical activity (HR: 1.916 in males, 2.102 in females), and waist circumference (HR: 1.019 in males, 1.026 in females) showed significant individual risks. The presence of hypertension (HR: 2.477 in males, 2.996 in females) and diabetes mellitus (HR: 2.331 in males, 2.307 in females) were the strongest individual predictors, more than doubling the risk of death in both groups.

**Table 3:** Sex-specific cox proportional hazards models for all-cause mortality: unadjusted and multivariable-adjusted analyses.

		Male				Females		
		HR	95 % Cl.	p-Value		HR	95 % Cl.	p-Value
Model 1	Age	1.089	1.081–1.098	<0.001	Age	1.098	1.089–1.107	<0.001
	Smoke-never	1			Smoke-never	1		
	Smoker-former	1.527	1.193–1.956	<0.001	Smoker-former	0.763	0.406–1.432	0.400
	Smoke-current	0.955	0.761–1.200	0.694	Smoke-current	0.538	0.383–0.757	<0.001
	Physical activity (low)	1.916	1.582–2.320	<0.001	Physical activity (low)	2.102	1.564–2.826	<0.001
	Income status- low	1			Income status- low	1		
	Income status- middle	0.541	0.420–0.697	<0.001	Income status- middle	0.540	0.398–0.734	<0.001
	Income status-high	0.478	0.375–0.608	<0.001	Income status-high	0.326	0.234–0.454	<0.001
	WC (cm)	1.019	1.010–1.027	<0.001	WC (cm)	1.026	1.018–1.034	<0.001
	HT (present)	2.477	2.056–2.984	<0.001	HT (present)	2.996	2.339–3.743	<0.001
	DM (present)	2.331	1.832–2.966	<0.001	DM (present)	2.307	1.774–3.001	<0.001
Model 2	Age	1.087	1.077–1.096	<0.001	Age	1.094	1.084–1.104	<0.001
	Smoke-never	1			Smoke-never	1		
	Smoker-former	1.339	1.045–1.716	0.021	Smoker-former	1.416	0.748–2.681	0.286
	Smoke-current	1.691	1.335–2.140	<0.001	Smoke-current	1.176	0.820–1.686	0.379
	Physical activity (low)	1.175	0.963–1.434	0.113	Physical activity (low)	1.117	0.823–1.517	0.477
	Income status- low	1			Income status- low	1		
	Income status- middle	0.946	0.727–1.230	0.678	Income status- middle	0.964	0.703–1.321	0.819
	Income status-high	0.743	0.580–0.952	0.019	Income status-high	0.705	0.495–1.004	0.053
	WC (cm)	1.002	0.993–1.011	0.662	WC (cm)	1.003	0.994–1.013	0.468
	HT (present)	1.538	1.264–1.872	<0.001	HT (present)	1.378	1.088–1.746	0.008
	DM (present)	1.583	1.235–2.030	<0.001	DM (present)	1.868	1.425–2.447	<0.001
Model 3 Total cholesterol, mg/dL	Q1	1			Q1	1		
	Q2	1.012	0.789–1.299	0.923	Q2	1.292	0.925–1.806	0.133
	Q3	0.924	0.710–1.202	0.554	Q3	1.289	0.926–1.794	0.133
	Q4	1.199	0.916–1.570	0.186	Q4	1.301	0.944–1.793	0.108
Model 4 HDL-C, mg/dL	Q1	1			Q1	1		
	Q2	0.862	0.659–1.127	0.279	Q2	1.025	0.638–1.645	0.919
	Q3	0.759	0.580–0.994	0.045	Q3	0.890	0.583–1.358	0.588
	Q4	0.863	0.661–1.126	0.278	Q4	1.050	0.713–1.546	0.806
Model 5 LDL-C, mg/dL	Q1	1			Q1	1		
	Q2	1.065	0.82–1.384	0.638	Q2	1.321	0.959–1.820	0.088
	Q3	0.826	0.632–1.078	0.160	Q3	0.968	0.705–1.330	0.842
	Q4	1.120	0.861–1.458	0.398	Q4	1.306	0.966–1.767	0.083
Model 6 Trigliceride, mg/dL	Q1	1			Q1	1		
	Q2	1.129	0.869–1.466	0.362	Q2	1.067	0.798–1.425	0.662
	Q3	1.116	0.845–1.474	0.441	Q3	1.143	0.842–1.550	0.392
	Q4	1.464	1.106–1.937	0.008	Q4	1.167	0.850–1.602	0.339

In the multivariate model (Model 2), which included age, smoking, physical activity, waist circumference, hypertension, and diabetes, age remained a consistent predictor (p<0.001). Hypertension (HR: 1.538 in males, p<0.001; HR: 1.378 in females, p=0.008) and diabetes mellitus (HR: 1.583 in males, p<0.001; HR: 1.868 in females, p<0.001) maintained their status as robust independent predictors. Notably, after mutual adjustment, the independent effects of low physical activity and waist circumference became non-significant in both sexes (p>0.05). Regarding smoking, current smoking remained a significant independent risk factor in males (HR: 1.691, p<0.001), whereas it did not show a significant independent association in females (p=0.379).

The independent contribution of lipid parameters was assessed by adding each lipid quartile separately to the multivariate Model 2 (Models 3–6). After adjusting for the covariates in Model 2, total cholesterol (Model 3), and LDL-C (Model 5) quartiles did not show a significant independent association with mortality in either sex (p>0.05 for all). In males, however, HDL-C showed a significant association in Model 4, where individuals in the third quartile (Q3) had a 24.1 % lower risk of all-cause mortality compared to those in the lowest quartile (Q1) (HR: 0.759, 95 % CI: 0.580–0.994, p=0.045). Additionally, in males, the highest triglyceride quartile (Q4) emerged as a significant independent predictor of all-cause mortality, associated with a 46.4 % increased risk compared to the lowest quartile (Model 6, HR: 1.464, 95 % CI: 1.106–1.937, p=0.008). No such independent associations for either HDL-C or triglycerides were observed in females.

### Non-linear associations with all-cause mortality

**Figure 2:**
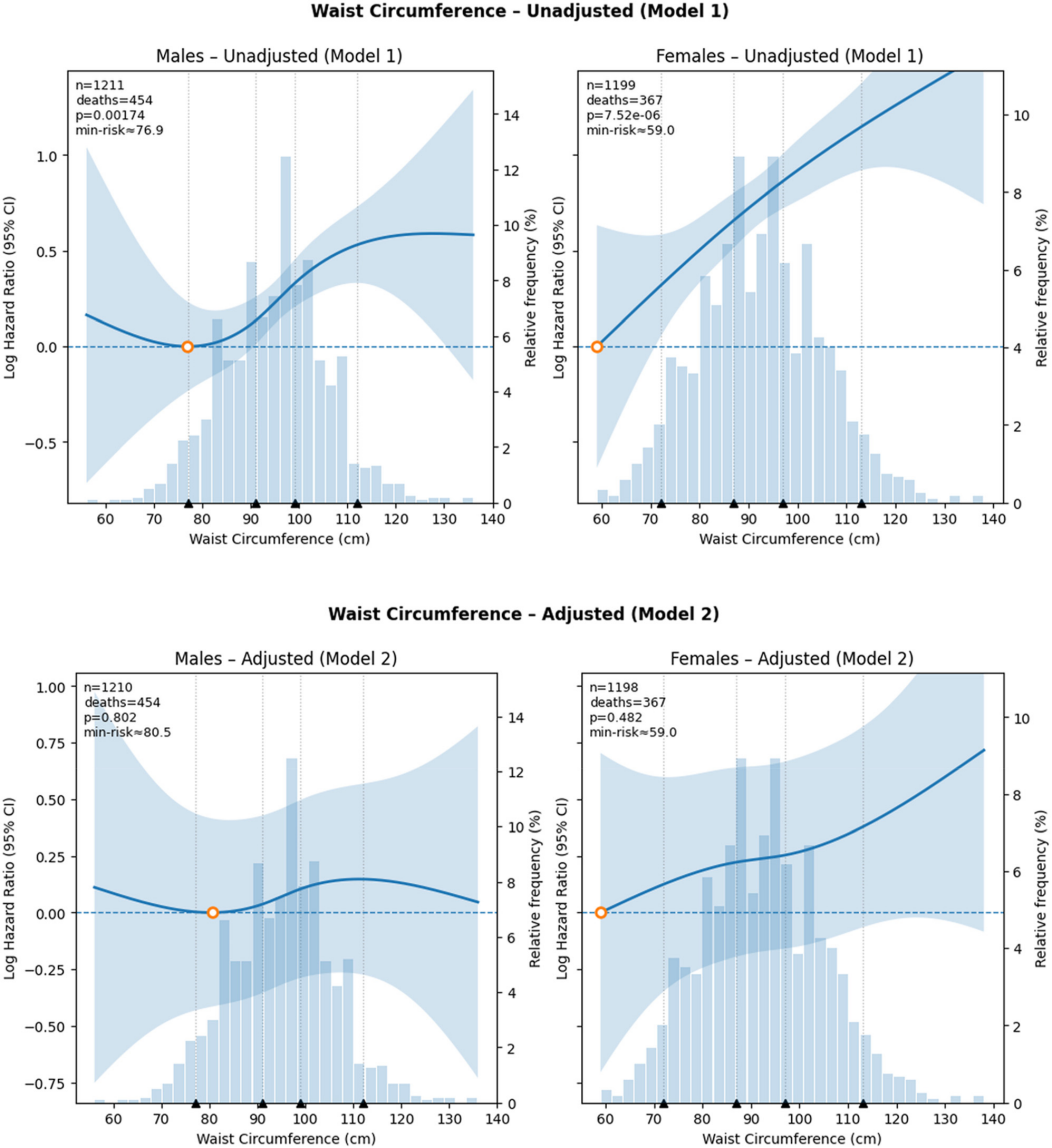
Restricted cubic spline (RCS) analysis of the association between waist circumference and all-cause mortality risk by sex. Note: Hazard ratios were estimated using cox proportional hazards regression, with waist circumference entered as a restricted cubic spline using 4 knots placed at the 5th, 35th, 65th, and 95th percentiles of the sex-specific waist circumference distribution; knot locations are indicated by vertical grey dashed lines and ▲ markers along the x-axis. The solid line is the point estimate of the log hazard ratio and the shaded band the 95 % confidence interval. The reference value (log HR=0; **o** marker) was set to the minimum predicted hazard.

**Figure 3:**
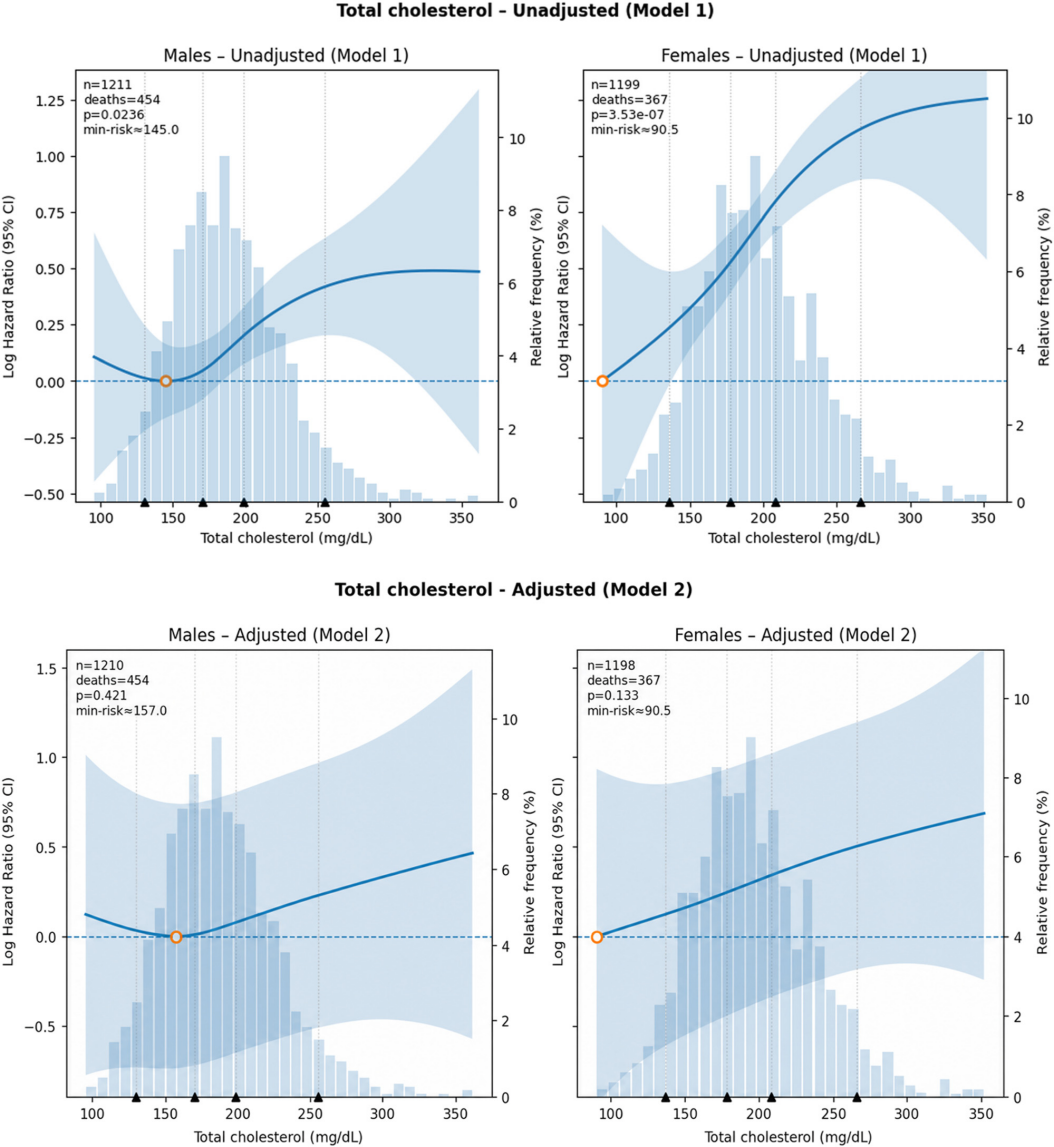
Restricted cubic spline (RCS) analysis of the association between total cholesterol levels and all-cause mortality risk by sex. Note: Hazard ratios were estimated using cox proportional hazards regression, with waist circumference entered as a restricted cubic spline using 4 knots placed at the 5th, 35th, 65th, and 95th percentiles of the sex-specific waist circumference distribution; knot locations are indicated by vertical grey dashed lines and ▲ markers along the x-axis. The solid line is the point estimate of the log hazard ratio and the shaded band the 95 % confidence interval. The reference value (log HR=0; **o** marker) was set to the minimum predicted hazard.

**Figure 4:**
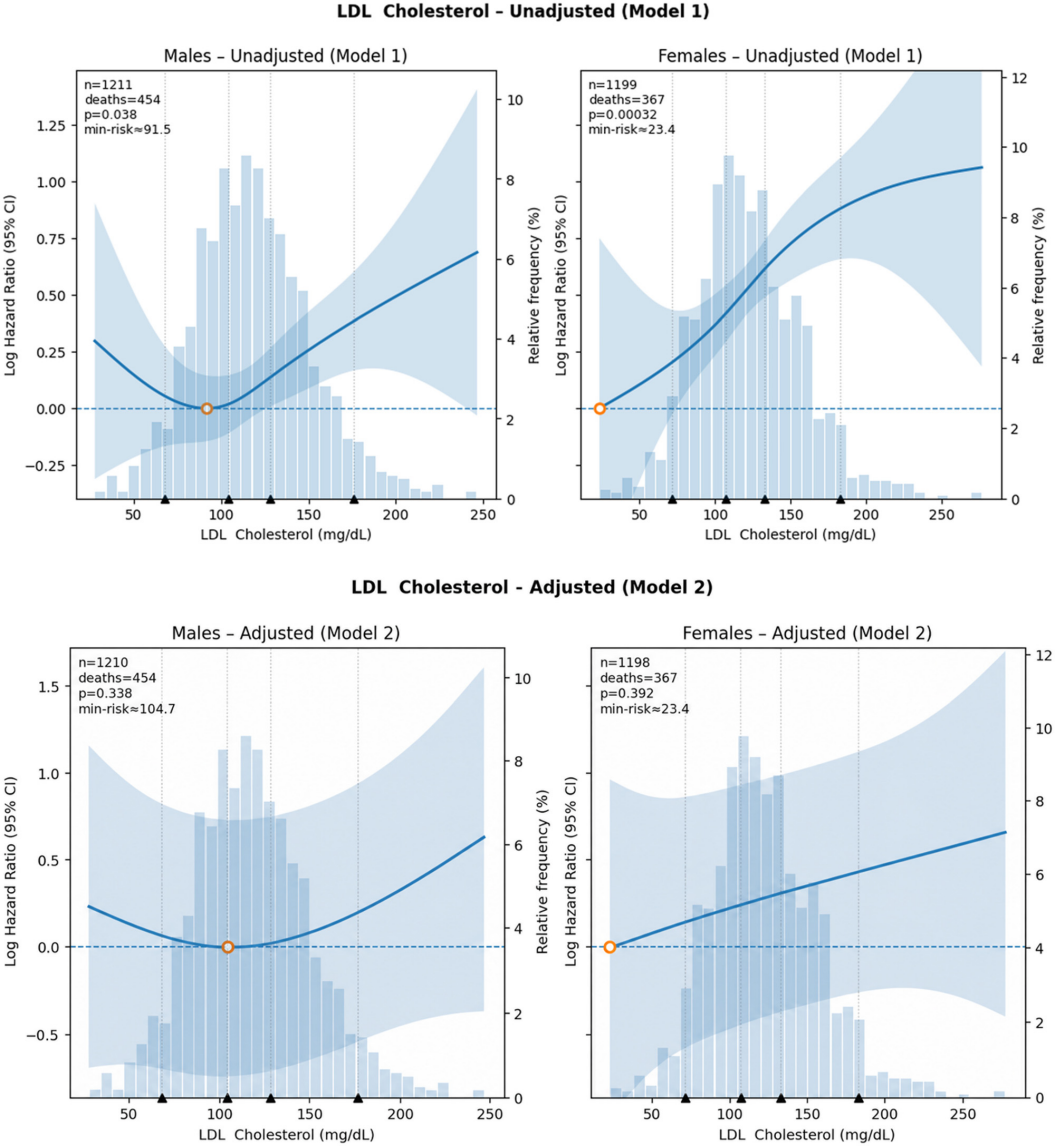
Restricted cubic spline (RCS) analysis of the association between LDL-Cholesterol levels and all-cause mortality risk by sex. Note: Hazard ratios were estimated using cox proportional hazards regression, with waist circumference entered as a restricted cubic spline using 4 knots placed at the 5th, 35th, 65th, and 95th percentiles of the sex-specific waist circumference distribution; knot locations are indicated by vertical grey dashed lines and ▲ markers along the x-axis. The solid line is the point estimate of the log hazard ratio and the shaded band the 95 % confidence interval. The reference value (log HR=0; **o** marker) was set to the minimum predicted hazard.

**Figure 5:**
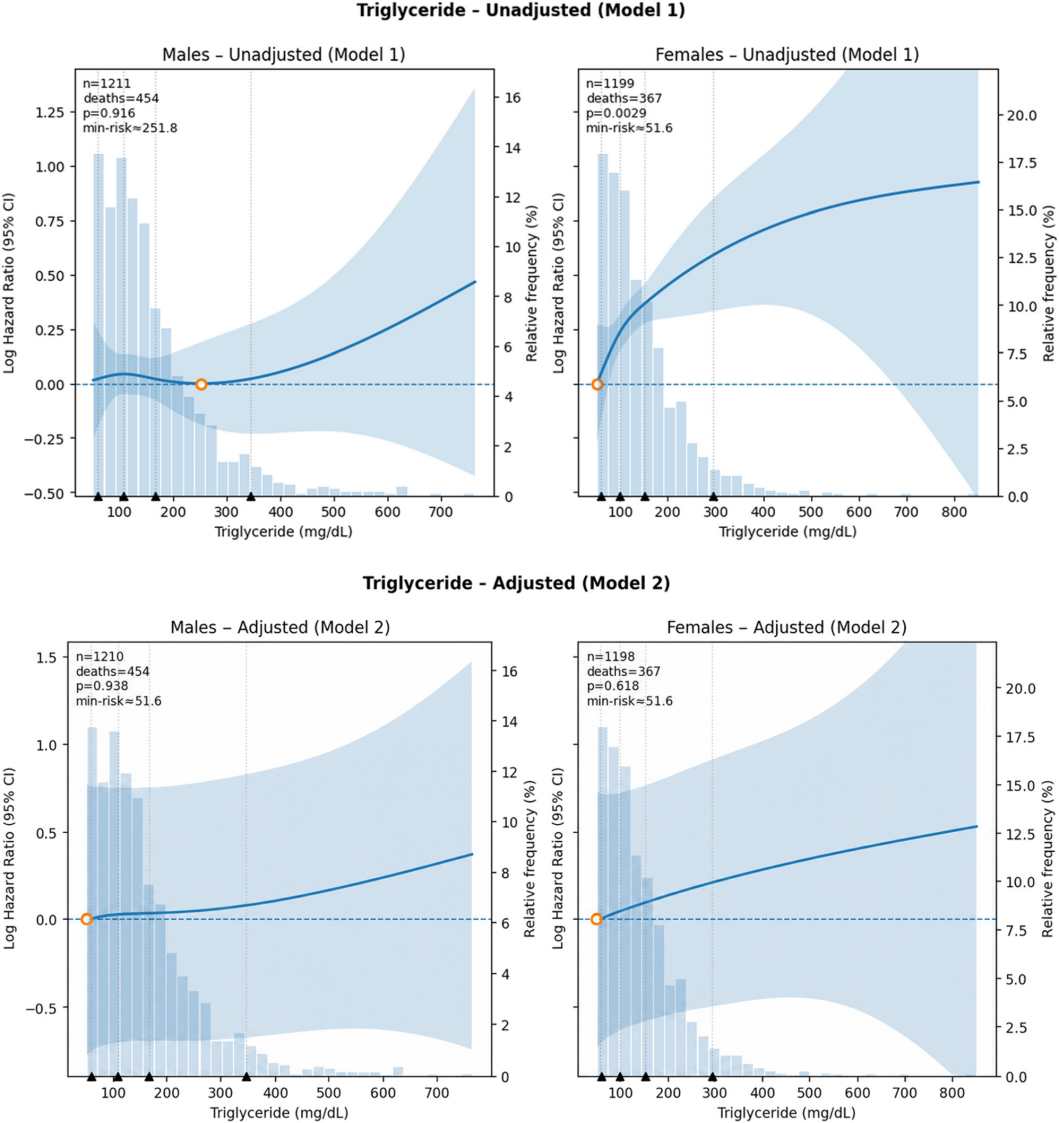
Restricted cubic spline (RCS) analysis of the association between triglyceride levels and all-cause mortality risk by sex. Note: Hazard ratios were estimated using cox proportional hazards regression, with waist circumference entered as a restricted cubic spline using 4 knots placed at the 5th, 35th, 65th, and 95th percentiles of the sex-specific waist circumference distribution; knot locations are indicated by vertical grey dashed lines and ▲ markers along the x-axis. The solid line is the point estimate of the log hazard ratio and the shaded band the 95 % confidence interval. The reference value (log HR=0; **o** marker) was set to the minimum predicted hazard.

**Figure 6:**
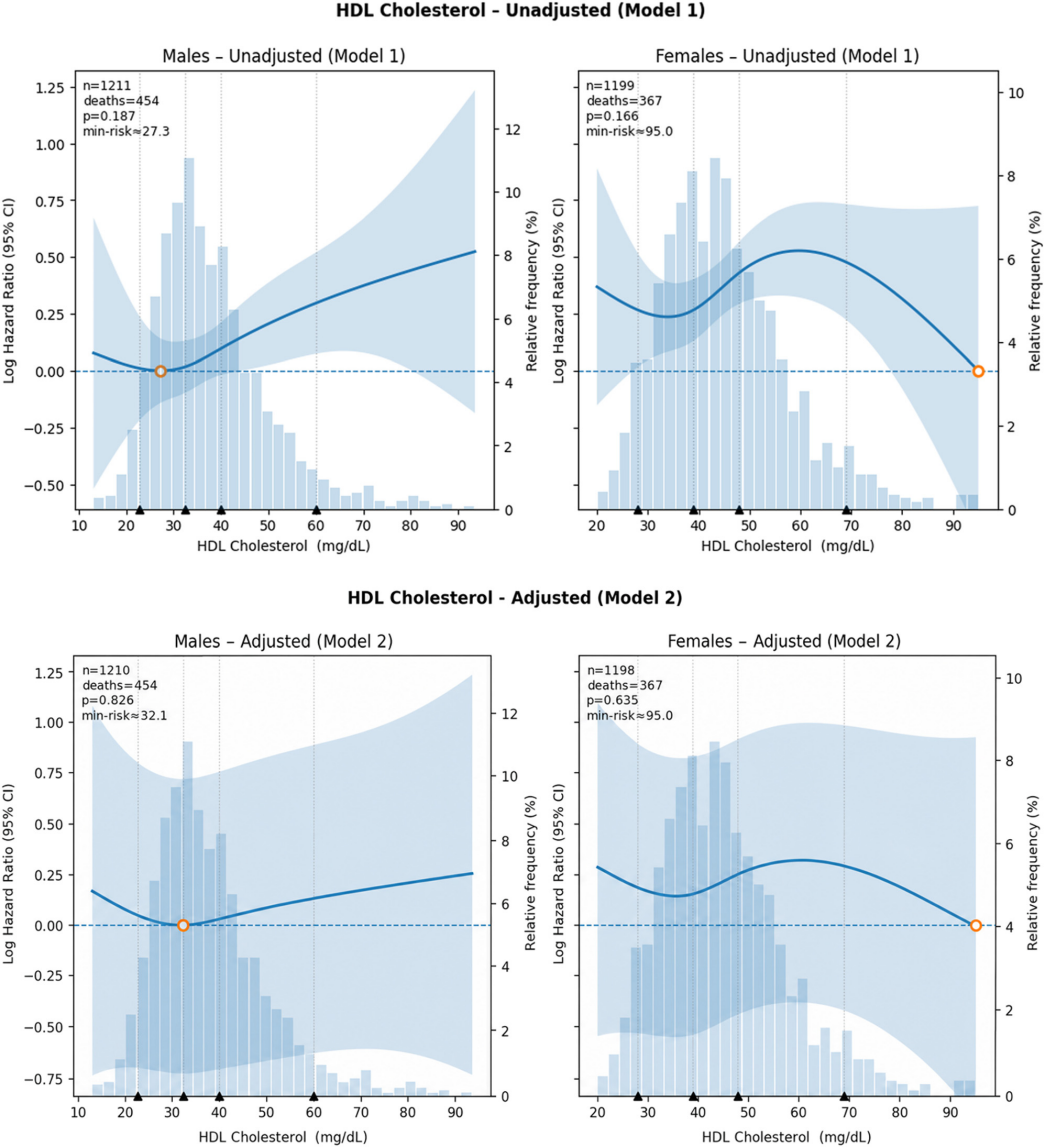
Restricted cubic spline (RCS) analysis of the association between HDL-Cholesterol levels and all-cause mortality risk by sex. Note: Hazard ratios were estimated using cox proportional hazards regression, with waist circumference entered as a restricted cubic spline using 4 knots placed at the 5th, 35th, 65th, and 95th percentiles of the sex-specific waist circumference distribution; knot locations are indicated by vertical grey dashed lines and ▲ markers along the x-axis. The solid line is the point estimate of the log hazard ratio and the shaded band the 95 % confidence interval. The reference value (log HR=0; **o** marker) was set to the minimum predicted hazard.

The non-linear relationships between continuous variables and all-cause mortality risk were evaluated using Restricted Cubic Spline (RCS) analysis (Table 4). In the unadjusted model (Model 1), significant non-linear associations were observed for waist circumference in both males (spline global p=0.002) and females (p<0.001), with the lowest mortality risk identified at 76.90 and 59.00 cm, respectively. Similarly, total cholesterol (min_risk_x: 144.95 mg/dL in males, 90.45 mg/dL in females) and LDL-cholesterol (min_risk_x: 91.52 mg/dL in males, 23.38 mg/dL in females) demonstrated significant non-linear associations with mortality in both sexes (p<0.05 for all). Triglyceride levels did not show a significant non-linear association with mortality in males (p=0.916), whereas a significant association was observed in females (p=0.003), with the minimum risk at 51.59 mg/dL (Figures 2–6).

**Table 4:** Non-linear associations between continuous variables and all-cause mortality: Restricted cubic spline analysis results.

Variable	Model	Sex	n	Deaths	min_risk_x	spline_global_p	used_penalizer	Knots
Waist circumference	Model1	Males	1,211	454	76.90	0.002	0.2	77.00, 91.00, 99.00, 112.00
Waist circumference	Model1	Females	1,199	367	59.00	<0.001	0.2	72.00, 87.00, 97.00, 113.00
Waist circumference	Model2	Males	1,210	454	80.52	0.802	0.3	77.00, 91.00, 99.00, 112.00
Waist circumference	Model2	Females	1,198	367	59.00	0.489	0.3	72.00, 87.00, 97.00, 113.00
Total cholesterol	Model1	Males	1,211	454	144.95	0.0246	0.2	130.00, 170.47, 198.97, 255.26
Total cholesterol	Model1	Females	1,199	367	90.45	<0.001	0.2	136.27, 177.76, 208.30, 265.93
Total cholesterol	Model2	Males	1,210	454	156.990	0.421	0.3	129.97, 170.43, 198.97, 255.31
Total cholesterol	Model2	Females	1,198	367	90.45	0.133	0.3	136.26, 177.76, 208.09, 265.93
HDL-cholesterol	Model1	Males	1,211	454	27.28	0.187	0.2	22.73, 32.40, 40.00, 60.00
HDL-cholesterol	Model1	Females	1,199	367	95.00	0.166	0.2	28.00, 38.91, 48.00, 69.00
HDL-cholesterol	Model2	Males	1,210	454	32.13	0.826	0.3	22.73, 32.40, 40.00, 60.00
HDL-cholesterol	Model2	Females	1,198	367	95.00	0.635	0.3	28.00, 38.91, 48.00, 69.00
LDL-cholesterol	Model1	Males	1,211	454	91.52	0.0038	0.2	67.82, 104.17, 128.02, 176.13
LDL-cholesterol	Model1	Females	1,199	367	23.38	<0.001	0.2	71.37, 107.12, 132.39, 182.54
LDL-cholesterol	Model2	Males	1,210	454	104.70	0.338	0.3	67.80, 104.16, 128.00, 176.16
LDL-cholesterol	Model2	Females	1,198	367	23.38	0.392	0.3	71.37, 107.11, 132.37, 182.56
Triglyceride	Model1	Males	1,211	454	251.79	0.916	0.2	59.95, 108.14, 166.03, 345.28
Triglyceride	Model1	Females	1,199	367	51.59	0.003	0.2	59.95, 98.88, 151.97, 294.96
Triglyceride	Model2	Males	1,210	454	51.59	0.938	0.3	59.95, 108.13, 165.92, 345.38
Triglyceride	Model2	Females	1,198	367	51.59	0.618	0.3	59.95, 98.84, 152.01, 295.01

Knot Percentiles: The knot points used in the analysis correspond to the 5th, 35th, 65th and 95th percentiles. Adjustment variables: Model 2 were adjusted for age, smoking, physical activity, hypertension, diabetes for waist circumference; waist circumference added for lipid profiles.

In the multivariate model (Model 2), which was adjusted for age, smoking, physical activity, hypertension, and diabetes, the global spline p-values for all evaluated parameters – including waist circumference, total cholesterol, HDL-C, LDL-C, and triglycerides – became non-significant in both males and females (p>0.05). Specifically, no significant non-linear associations were observed for triglycerides in either males (p=0.938) or females (p=0.618), nor for HDL-C in males (p=0.826) or females (p=0.635). This indicates that the non-linear associations observed in the unadjusted models were largely attenuated after controlling for traditional cardiovascular risk factors, particularly age, hypertension, and diabetes mellitus (Figures 2–6).

## Discussion

The aim of this study was to investigate the relationships between lipid profiles, including total cholesterol, LDL-C, HDL-C and triglyceride levels, and anthropometric measurements and their effects on all-cause mortality rates in a Turkish cohort database. Although most lipid parameters did not demonstrate independent associations with all-cause mortality after adjustment for established risk factors, elevated triglyceride levels in the highest quartile emerged as a significant independent predictor of mortality in males, representing an important sex-specific finding. In addition, higher HDL-C levels (third quartile) were associated with a reduced risk of mortality in males. Age, former smoking, hypertension and diabetes mellitus were consistently associated with all-cause mortality across both sexes.

In our study, elevated levels of total cholesterol and LDL-C were indeed associated with increased all-cause mortality in univariate analyses. Additionally, in females, triglyceride levels were significantly higher in the mortality group. In females, a significantly elevated mortality rate was observed in those within the fourth quartile (Q4) of low-density lipoprotein cholesterol (LDL-C) levels compared with the first quartile (Q1) and second quartile (Q2), with a similarly higher mortality rate in the fourth quartile (Q4) of triglyceride levels than in Q1, and the lowest mortality rate was recorded among those in the first quartile (Q1) of total cholesterol levels. However, when adjusting for key risk factors such as age, smoking, hypertension, and diabetes, these associations lost their statistical significance.

In males, the fourth quartile (Q4) of triglycerides was linked to a markedly higher mortality risk compared to the first quartile (Q1) (HR: 1.464; 1.106–1.937); this noteworthy sex-specific association may be rooted in several biological and behavioral mechanisms. This finding indicates that while lipid levels may influence mortality risk, their impact is likely mediated or overshadowed by stronger predictors, possibly showing a complex relationship, particularly in the context of other established risk factors. Wu et al. concluded that even when classified as ‘optimal’ or ‘normal’ by traditional guidelines, TG levels serve as a significant predictor of long-term all-cause mortality. This finding reinforces the clinical importance of monitoring TG levels as a biomarker of all cause mortality risk [14]. Biologically, sex hormones fundamentally dictate lipid metabolism and adipose tissue distribution [15], 16]. Estrogens enhance hepatic clearance of circulating lipids and favor beneficial lipoprotein profiles, potentially mitigating the atherogenic potential of elevated triglycerides in females [16]. Conversely, males typically exhibit higher hepatic lipase activity which promotes the formation of highly atherogenic small dense LDL particles. Furthermore, males are genetically predisposed to visceral adiposity, which is deeply interlinked with systemic insulin resistance, localized inflammation, and an increased release of pro-thrombotic factors [17]. High triglyceride levels in the context of visceral adiposity create a highly pro-inflammatory and pro-thrombotic milieu. Behavioral factors in our Turkish cohort likely synergize with these physiological vulnerabilities: baseline data show markedly higher prevalence of current smoking (48.1 vs. 16.2 %) and alcohol use (18.0 vs. 0.8 %) among males compared with females. Alcohol directly stimulates hepatic triglyceride synthesis, while smoking exacerbates oxidative stress, endothelial dysfunction, and systemic inflammation [18]. It is highly plausible that the interaction between visceral adiposity, accelerated atherogenic particle formation, and concurrent high-risk behaviors creates a synergistic effect, amplifying the mortality risk associated with hypertriglyceridemia specifically in males. However, we must explicitly acknowledge that our findings demonstrate an independent observational association rather than direct causality. The complex and potentially non-linear dynamics of lipid-mortality relationships require that these male-specific findings be interpreted cautiously. Future investigations with longitudinal lipid tracking and specific biomarker profiling in independent, large-scale cohorts are necessary to replicate these findings and definitively elucidate the underlying pathophysiological pathways.

Studies investigating the impact of lipid profiles on all-cause mortality have identified distinct non-linear patterns. Specifically, a U-shaped relationship has been demonstrated between LDL-C levels and mortality risk [5], a pattern also observed for HDL-C levels in both males and females [19], 20]. Additionally, research has characterized the association between all-cause mortality and levels of LDL-C, HDL-C, and total cholesterol as following a J-shaped trajectory [21]. Although the J- or U-shaped curve frequently discussed in the literature regarding the relationship between HDL-C and all-cause mortality was not clearly observed in our study, we found that the HDL-C Q3 level was associated with a 24.1 % reduction in all cause mortality in males, whereas no such significant association was observed in females. (Males, HR: 0.759, 95 % CI: 0.580–0.994, p=0.045; Females, HR: 0.890, 95 % CI: 0.583–1.358, p=0.588).

A study conducted by Xu et al. identified systolic blood pressure, BMI, LDL-C, fasting blood glucose and smoking status as key risk factors. Effective management of these factors was found to significantly reduce the incidence of all-cause mortality and heart failure in both diabetic patients and healthy controls [22].

Another study confirmed that elevated LDL-C and total cholesterol levels were correlated with an increased risk of all-cause mortality in univariate analyses, but after further adjustment, the third quartile of TC was related to an increased risk of all-cause mortality and an inverse relationship between triglyceride (TG) and LDL-C levels. Specifically, participants in the second quartile of TG and third quartile of LDL-C had a lower mortality risk [23].

Similarly to this study, Huyang Y. et al. reported that triglyceride levels are associated with all-cause and cardiovascular mortality via univariate analysis; however, elevated triglycerides were not statistically significant for cardiovascular mortality when they were adjusted for age, sex, smoking status, BMI, SBP, and other essential factors [8].

In this study, the RCS analysis provided insight into the nature of the associations between anthropometric and lipid parameters and mortality observed in our cohort. For waist circumference, the minimum mortality risk thresholds identified in our cohort (approximately 76.90 cm in males and 59.00 cm in females) were lower than those reported in the international literature. A meta-analysis of 29 cohort studies including over 58,000 participants aged 65–74 identified a J-shaped relationship with all-cause mortality, with the nadir at 94 cm in males and 77 cm in females [24]. Although total cholesterol (minimum risk thresholds of 144.95 mg/dL for males and 90.45 mg/dL for females) and LDL-C showed significant non-linear relationships with mortality in unadjusted models, these associations disappeared after adjusting for age, hypertension, and diabetes. The total cholesterol threshold for males is concordant with Johannesen et al. who reported the lowest all-cause mortality risk at 140 mg/dL in the general population and at 89 mg/dL among individuals receiving lipid-lowering treatment – closely paralleling our female threshold [5]. For LDL-C, Liu et al. reported a U-shaped association with all-cause mortality in fully adjusted models, with the lowest mortality risk at approximately 130 mg/dL [25]. HDL-C showed no significant nonlinear association even in unadjusted models, despite minimum risk thresholds of 27.28 mg/dL in males and 95.00 mg/dL in females. The sex-specific nonlinear pattern observed for triglycerides in females (Model 1 p=0.003, minimum risk threshold: 51.59 mg/dL) also lost significance after adjustment.

In this study, both male and female sex, hypertension (HR: 1.538:1.264–1.872 in males; HR: 1.378:1.088–1.746 in females) and diabetes mellitus (HR: 1.583:1.235–2.030 in males; HR: 1.868:1.425–2.447 in females) were found to be independent risk factors for all-cause mortality. Numerous studies have shown that hypertension and diabetes are major risk factors for all-cause mortality. Hypertension and diabetes mellitus have been well documented as independent predictors of increased mortality risk in both males and females [26], [27], [28].

The relationship between smoking and mortality in our study revealed an apparent paradoxical inverse association in univariate analyses for both sexes. However, age-stratified analyses demonstrated this was entirely due to age confounding. After multivariate adjustment, the true detrimental effects emerged: both current smoking (HR: 1.691, 95 % CI: 1.335–2.140, p<0.001) and former smoking (HR: 1.339, 95 % CI: 1.045–1.716, p=0.021) were independently associated with increased mortality in males. In females, no significant association was detected with former smoking (HR: 1.416, 95 % CI: 0.748–2.681, p=0.286), nor was there a significant association with current smoking (HR: 1.176, 95 % CI: 0.820–1.686, p=0.379), likely due to the low number of female smokers. These findings align with established evidence that smoking increases mortality in both sexes [29], [30], [31].

A prospective cohort study in Japan confirmed that smoking is associated with an increased risk of death from all causes as well as all cancers, cardiovascular diseases and respiratory diseases in both sexes, suggesting that smoking is responsible for a significant proportion of all-cause deaths as well as cause-specific deaths [32]. The TEKHARF cohort study of Turkish females published in 2007 revealed that a history of smoking in females was a protective factor against metabolic syndrome and obesity, but smoking had no effect on mortality in females [33].

A meta-analysis of 72 prospective cohort studies revealed a J-shaped association between waist circumference and waist-to-height ratio and the risk of all-cause mortality in both males and females [10]. Studies suggest the use of waist circumference or the waist‒to-hip ratio in addition to BMI in the assessment of mortality risk [34], [35], [36]. Similar to previous studies, we found that the mean waist circumference was significantly greater in patients who died during follow-up, whereas the differences BMI between the deceased and living groups were not significant in either sex. Also in our study, in males, there was no statistically significant difference in hip circumference measurements between the mortality and survival groups. Although in females, the mortality group has significantly higher hip circumference values. However, we did not observe this effect in multivariate analyses, suggesting that the influence of waist circumference may be mediated by other factors in the model, such as hypertension and diabetes.

In both sexes, participants who deceased had greater systolic and diastolic blood pressure values. Similar to our findings, a study by Xu et al. revealed that the prevalence of all-cause mortality increased in parallel with SBP [22].

In our findings, the mortality rate was higher in the group with low physical activity in both males and females. A study examining the association of physical activity with all-cause mortality in both sexes reported results similar to our findings and additionally reported that equivalent amounts of leisure-time physical activity reduced the risk of all-cause mortality more for females than for males [37].

In this study, the highest mortality rate in both sexes was found in the low-income group. Similar to our findings, in the Gutenberg Health Study with 4,084 participants followed up for 10 years, low socioeconomic status was found to be associated with mortality [38].

This study has several strengths and limitations that should be acknowledged. A major strength is the use of long-term follow-up data from the TEKHARF cohort, which provided a robust basis for longitudinal analysis. The 25-year follow-up duration, prospective design, and comprehensive sex-stratified analytical approach using restricted cubic spline regression provide valuable insights into lipid-mortality associations in this understudied Turkish population. However, certain limitations warrant consideration. First, lipid measurements and behavioral risk factors (smoking, alcohol consumption, and physical activity) were obtained only at baseline and were not reassessed during follow-up, which precludes evaluation of temporal changes over the 25-year observation period. Furthermore, the risk factor associations reflect baseline conditions and do not account for changes in antihypertensive, glycemic, and lipid-lowering management or broader shifts in preventive care during follow-up. Smoking status was recorded categorically without pack-year quantification, and physical activity was assessed using a binary classification. These single-assessment measures may result in exposure misclassification and fail to capture lifestyle changes over time. Second, diabetes staging data (e.g., duration, severity, glycemic control, microvascular complications) were not available for all participants, which limits our ability to precisely characterize diabetes-related mortality risk. Third, family history of cardiovascular disease was not assessed. Fourth, despite adjustment for multiple cardiovascular risk factors, potential residual confounding remains. We lacked data on dietary patterns and detailed socioeconomic variables beyond income category, both of which may independently influence lipid profiles and mortality. Fifth, the generally low HDL-cholesterol levels across the population might have constrained the statistical power of quartile-based analyses for this parameter. Finally, some participants had missing data for certain biochemical, anthropometric, sociodemographic, or clinical variables. To preserve sample size and statistical power, these cases were retained, leading to slight discrepancies in the total number of measurements for each specific parameter. Additionally, our findings are derived from a Turkish adult population and may not be directly generalizable to populations with different genetic backgrounds, lifestyle patterns, or healthcare systems.

## Conclusions

Our findings indicate that although total cholesterol, LDL-C, and triglyceride levels were associated with all-cause mortality in unadjusted analyses, these associations were largely attenuated after adjustment for established cardiometabolic risk factors. Age, hypertension and diabetes mellitus emerged as the most consistent and robust independent predictors of mortality in both sexes. Notably, elevated triglyceride levels remained an independent predictor of mortality in males, highlighting a potential sex-specific effect that warrants further investigation. In addition, higher HDL-C levels (specifically in the third quartile) were associated with a reduced risk of all-cause mortality in males, further supporting the sex-specific nature of lipid-mortality associations. In males, the consistency of the association between lipids levels and all-cause mortality with the existing literature supports its biological plausibility and underscores the importance of monitoring triglycerides and HDL-C as part of a comprehensive risk assessment. It should be acknowledged, however, that this finding needs to be replicated in independent cohorts to establish causal relationships.

These results suggest that lipid parameters alone may not be sufficient predictors of long-term survival when considered outside the broader cardiometabolic context. Comprehensive risk assessment models incorporating traditional clinical risk factors remain essential for mortality prediction. Future studies should focus on longitudinal lipid trajectories, potential reverse causality, and sex-specific mechanisms underlying triglyceride- and HDL-C- associated mortality risk.

## Supplementary Material

Supplementary Material
