# Supplementary material for: Impact of lipid profiles and risk factors on all-cause mortality in males and females: insights from a Turkish cohort study
Source: Open Med (Wars). 2026 Aug 6;21(1):20261504. doi: 10.1515/med-2026-1504 (PMC13439538; doi:10.1515/med-2026-1504)
Supplement: Supplementary file 1 [file j_med-2026-1504_suppl_001.docx]

Supplementary Table 1: Baseline Characteristics of the Study Population by Sex.

|  | **Males** | **Females** | **p-value** |  |
| --- | --- | --- | --- | --- |
| **Income (%)** |  | | |  |
| **Low** | 626 (51.7%) | 709 (59.1%) | **0.001 ^ж^** |  |
| **Middle** | 259 (21.4%) | 218 (18.2%) |  |  |
| **High** | 326 (26.9%) | 272 (22.7%) |  |  |
| **Smoke** |  | | |  |
| **Never** | 342 (28.2%) | 957 (79.8%) | **<0.001 ^ж^** |  |
| **Former** | 287 (23.7%) | 48 (4%) |  |  |
| **Current** | 582 (48.1%) | 194 (16.2%) |  |  |
| **Alcohol User** |  | | |  |
| **No** | 993 (82%) | 1189 (99.2%) | **<0.001 ^ж^** |  |
| **Yes** | 218 (18%) | 10 (0.8%) |  |  |
| **Physical Activity** |  | | |  |
| **Low** | 613 (50.6%) | 895 (74.6%) | **<0.001 ^ж^** |  |
| **More Active** | 598 (49.4%) | 304 (25.4%) |  |  |
| **Hypertension** | | | | |
| **Absent** | 738 (60.9%) | 628 (52.4%) | **<0.001 ^ж^** |  |
| **Present** | 473 (39.1%) | 571 (47.6%) |  |  |
| **Diabetes Mellitus** | | | | |
| **Absent** | 1075 (88.8%) | 1060 (88.4%) | 0.780 ^ж^ |  |
| **Present** | 136 (11.2%) | 139 (11.6%) |  |  |
| **Mortality** |  | | |  |
| **Absent** | 757 (62.5%) | 832 (69.4%) | **<0.001 ^ж^** |  |
| **Present** | 454 (37.5%) | 367 (30.6%) |  |  |
| **Age (Years)** | 52 (22-96) | 52 (31-99) | 0.292 ^м^ |  |
| **Waist Circumference (cm)** | 95.01±10.78 | 92.32±12.61 | **<0.001 ^ϯ^** |  |
| **Hip Circumference (cm)** | 101.21±7.76 | 107.89±11.13 | **<0.001 ^ϯ^** |  |
| **BMI** | 26.79 (14.33-42.67) | 29.09 (16.53-53.96) | **<0.001 ^м^** |  |
| **SBP (mmHg)** | 125 (80-247) | 130 (80-250) | **<0.001 ^м^** |  |
| **DBP (mmHg)** | 80 (50-147.5) | 81 (48-170) | **<0.001 ^м^** |  |
| **Total Cholesterol (mg/dL)*** | 187.81±38.7 184.92 (95.48-361.58) | 195.94±40.36 192.93 (90.45-352) | **<0.001 ^ϯ^** |  |
| **HDL** **Cholesterol (mg/dL)*** | 37.96±11.73 35.64 (13.13-93.55) | 45.1±12.39 43.68 (20-95) | **<0.001 ^ϯ^** |  |
| **LDL Cholesterol (mg/dL)** | 115.8 (27.79-246.45) | 119.59 (23.38-276.42) | **0.003 ^м^** |  |
| **Triglyceride (mg/dL)** | 134.05 (51.59-763) | 120.84 (51.59-850) | **<0.001 ^м^** |  |
| “*”Log transform  ж: Pearson’s Chi Square Test  м:Mann Whitney U Test  ϯ:Independent Samples of T test | | | | |

Supplementary Table 2: Baseline Characteristics Stratified by Age Group (≤52 vs >52 years). Sex. and Mortality Status

|  | **Males** | | | | | | **Females** | | | | | |
| --- | --- | --- | --- | --- | --- | --- | --- | --- | --- | --- | --- | --- |
|  | **Age≤52 years** | | | **Age> 52 years** | | | **Age≤52 years** | | | **Age> 52 years** | | |
|  | **Alive** | **Death** | **p** | **Alive** | **Death** | **p** | **Alive** | **Death** | **p** | **Alive** | **Death** | **p** |
| **Income** | | | | | | | | | | | | |
| **Low** | 215 (85.3%) | 37 (14.7%) | 0.597 ^ж^ | 119 (31.9%) | 254 (68.1%)^a^ | **0.002** ^ж^ | 263 (91%) | 26 (9%) | 0.142 ^ж^ | 166 (39.6%) | 253 (60.4%)^a^ | **<0.001** ^ж^ |
| **Middle** | 139 (84.8%) | 25 (15.2%) |  | 43 (45.3%) | 52 (54.7%)^b^ |  | 115 (90.6%) | 12 (9.4%) |  | 55 (60.4%) | 36 (39.6%)^b^ |  |
| **High** | 184 (88%) | 25 (12%) |  | 56 (47.9%) | 61 (52.1%)^b^ |  | 202 (95.3%) | 10 (4.7%) |  | 30 (50%) | 30 (50%)^a.b^ |  |
| **Smoke Habits** | | | | | | | | | | | | |
| **Never** | 144 (97.3%) | 4 (2.7%)^a^ | **<0.001** ^ж^ | 79 (40.7%) | 115 (59.3%) | 0.425 ^ж^ | 417 (91.9%) | 37 (8.1%) | 0.621 ^ж^ | 219 (43.6%) | 283 (56.4%) | 0.603 ^ж^ |
| **Former** | 90 (83.3%) | 18 (16.7%)^b^ |  | 61 (34.3%) | 117 (65.7%) |  | 27 (96.4%) | 1 (3.6%) |  | 11 (55%) | 9 (45%) |  |
| **Current** | 304 (82.4%) | 65 (17.6%)^b^ |  | 78 (36.6%) | 135 (63.4%) |  | 136 (93.2%) | 10 (6.8%) |  | 21 (43.8%) | 27 (56.3%) |  |
| **Alcohol Consumption** | | | | | | | | | | | | |
| **No** | 425 (88%) | 58 (12%) | **0.011** ^ж^ | 193 (37.9%) | 316 (62.1%) | 0.398 ^ж^ | 573 (92.4%) | 47 (7.6%) | 0.473^Ғ^ | 251 (44.2%) | 317 (55.8%) | 0.506 ^Ғ^ |
| **Yes** | 113 (79.6%) | 29 (20.4%) |  | 25 (32.9%) | 51 (67.1%) |  | 7 (87.5%) | 1 (12.5%) |  | 0 (0%) | 2 (100%) |  |
| **Physical Activity** | | | | | | | | | | | | |
| **Low** | 209 (85.3%) | 36 (14.7%) | 0.654 ^ж^ | 116 (31.5%) | 252 (68.5%) | **<0.001** ^ж^ | 382 (92%) | 33 (8%) | 0.685 ^ж^ | 197 (41%) | 283 (59%) | **<0.001** ^ж^ |
| **More Active** | 329 (86.6%) | 51 (13.4%) |  | 102 (47%) | 115 (53%) |  | 198 (93%) | 15 (7%) |  | 54 (60%) | 36 (40%) |  |
| **Hypertension** | | | | | | | | | | | | |
| **Absent** | 410 (89.3%) | 49 (10.7%) | **<0.001** ^ж^ | 128 (45.9%) | 151 (54.1%) | **<0.001** ^ж^ | 408 (93.6%) | 28 (6.4%) | 0.083 ^ж^ | 107 (55.7%) | 85 (44.3%) | **<0.001** ^ж^ |
| **Present** | 128 (77.1%) | 38 (22.9%) |  | 90 (29.4%) | 216 (70.6%) |  | 172 (89.6%) | 20 (10.4%) |  | 144 (38.1%) | 234 (61.9%) |  |
| **Diabet Mellitus** | | | | | | | | | | | | |
| **Absent** | 508 (86.4%) | 80 (13.6%) | 0.365 ^ж^ | 194 (39.9%) | 292 (60.1%) | **0.003** ^ж^ | 544 (93.3%) | 39 (6.7%) | **0.004**^Ғ^ | 217 (45.6%) | 259 (54.4%) | 0.093 ^ж^ |
| **Present** | 30 (81.1%) | 7 (18.9%) |  | 24 (24.2%) | 75 (75.8%) |  | 36 (80%) | 9 (20%) |  | 34 (36.2%) | 60 (63.8%) |  |
| **Waist Circumference (cm)** | 93.14±10.54 | 95.32±12 | 0.080 ϯ | 95.91±10.25 | 97.09±10.72 | 0.191ϯ | 88 (61-125) | 94.5 (63-138) | **0.012 ^м^** | 95.87±11.95 | 95.54±12.12 | 0.741ϯ |
| **Hip Circumference (cm)** | 100.7±7.63 | 101.55±8.3 | 0.341 ϯ | 102 (84-126) | 102 (82-130) | 0.913 ^м^ | 105.5 (75-148) | 109 (88-152) | 0.174 ^м^ | 109 (80-146) | 108 (84-144) | 0.323 ^м^ |
| **BMI** | 26.57 (14.87-42.67) | 27.06 (18.37-39.06) | 0.230 ^м^ | 27.1 (18.38-42.61) | 26.89 (14.33-39.42) | 0.818 ^м^ | 28.31 (16.66-50.95) | 31.35 (20.83-53.96) | **0.004 ^м^** | 30.3 (16.53-50.08) | 29.33 (17.09-48.44) | **0.042 ^м^** |
| **SBP (mmHg)** | 120 (80-200) | 128 (90-235) | **<0.001 ^м^** | 128 (89-207) | 138 (85-247) | **<0.001 ^м^** | 121 (80-221) | 130 (90-250) | 0.005 ^м^ | 135 (82-250) | 150 (81-249) | **<0.001 ^м^** |
| **DBP (mmHg)** | 79 (50-130) | 81 (60-147.5) | **0.034^м^** | 80 (55-124) | 82 (50-131) | 0.105 ^м^ | 80 (48-130) | 80 (60-170) | 0.492 ^м^ | 84 (53-128) | 87 (60-134) | **0.015 ^м^** |
| **Total Cholesterol (mg/dL)** | 183.4±38.64 177.82 (102.51-343.48) | 194±43.4 190.89 (95.95-361.58) | **0.030* ϯ** | 187.63 (105.04-309.54) | 187 (95.48-358.61) | 0.748 ^м^ | 180.58 (103.02-352) | 193.22 (113.22-266.2) | 0.040 ^м^ | 205 (95.95-340.91) | 200.99 (90.45-349.65) | 0.935 ^м^ |
| **HDL Cholesterol (mg/dL)** | 37.79±13.56 34.04 (17.17-81.22) | 37.79±13.56 34.04 (17.17-81.22) | 0.749 *ϯ | 38.98±11.28  187.63 (105.04-309.54) | 38.98±12.2  187 (95.48-358.61) | 0.814*ϯ | 44.27±12.67 42.38 (20-94.84) | 41.67±12.06 38.49 (23-71.98) | 0.134* ϯ | 46.2±12.48 44.06 (25-95) | 46.2±11.67 45.51 (21.92-92.15) | 0.930* ϯ |
| **LDL Cholesterol (mg/dL)** | 110.85 (37.01-226.8) | 118.46 (37.17-213.87) | 0.253 ^м^ | 122.87 (28.46-199.27) | 118.66 (27.79-246.45) | 0.355 ^м^ | 111.51 (23.38-249.74) | 123.48 (48.73-166.16) | 0.060 ^м^ | 128.28 (36.66-276.42) | 125.78 (45-272.9) | 0.810 ^м^ |
| **Triglyceride (mg/dL)** | 138.47 (51.59-763) | 164.3 (62.7-553.72) | **0.003 ^м^** | 131.95 (53.8-680.4) | 118.81 (53-637) | 0.904 ^м^ | 110.05 (52-850) | 128.17 (55-419) | **0.015 ^м^** | 129.71 (58.8-542.64) | 130.8 (51.59-704.35) | 0.989 ^м^ |
| *logaritmik  ж: Pearson’s Chi Square Test  м:Mann Whitney U Test  ϯ:Independent Samples of T test  Ғ:Fisher’s Exact Test | | | | | | | | | | | | |
